# Insight of Captagon Abuse by Chemogenomics Knowledgebase-guided Systems Pharmacology Target Mapping Analyses

**DOI:** 10.1038/s41598-018-35449-6

**Published:** 2019-02-19

**Authors:** Nan Wu, Zhiwei Feng, Xibing He, William Kwon, Junmei Wang, Xiang-Qun Xie

**Affiliations:** 1 Department of Pharmaceutical Sciences and Computational Chemical Genomics Screening Center, School of Pharmacy, University of Pittsburgh, Pittsburgh, Pennsylvania 15261 United States; 2 National Center of Excellence for Computational Drug Abuse Research, University of Pittsburgh, Pittsburgh, Pennsylvania 15261 United States; 3 Drug Discovery Institute, University of Pittsburgh, Pittsburgh, Pennsylvania 15261 United States; 4 Departments of Computational Biology and Structural Biology, School of Medicine, University of Pittsburgh, Pittsburgh, Pennsylvania 15261 United States

## Abstract

Captagon, known by its genetic name Fenethylline, is an addictive drug that complicates the War on Drugs. Captagon has a strong CNS stimulating effect than its primary metabolite, Amphetamine. However, multi-targets issues associated with the drug and metabolites as well as its underlying mechanisms have not been fully defined. In the present work, we applied our established drug-abuse chemogenomics-knowledgebase systems pharmacology approach to conduct targets/off-targets mapping (SP-Targets) investigation of Captagon and its metabolites for hallucination addiction, and also analyzed the cell signaling pathways for both Amphetamine and Theophylline with data mining of available literature. Of note, Amphetamine, an agonist for trace amine-associated receptor 1 (TAAR1) with enhancing dopamine signaling (increase of irritability, aggression, etc.), is the main cause of Captagon addiction; Theophylline, an antagonist that blocks adenosine receptors (e.g. A2aR) in the brain responsible for restlessness and painlessness, may attenuate the behavioral sensitization caused by Amphetamine. We uncovered that Theophylline’s metabolism and elimination could be retarded due to competition and/or blockage of the CYP2D6 enzyme by Amphetamine; We also found that the synergies between these two metabolites cause Captagon’s psychoactive effects to act faster and far more potently than those of Amphetamine alone. We carried out further molecular docking modeling and molecular dynamics simulation to explore the molecular interactions between Amphetamine and Theophylline and their important GPCRs targets, including TAAR1 and adenosine receptors. All of the systems pharmacology analyses and results will shed light insight into a better understanding of Captagon addiction and future drug abuse prevention.

## Introduction

Captagon, the trademark name for the synthetic stimulant Fenethylline^1–6^, was first reported by a German pharmacist in 1961 for the potential treatment of hyperactivity, depression and narcolepsy^1^. However, due to its addictive and hallucinogenic properties, it was listed as a controlled substance by the United States (1981) and the World Health Organization (1986), making it illegal to buy or sell Captagon in most countries. Captagon has been reported to be a central nervous system (CNS) stimulator with stronger and longer lasting effects on fighting aggression, detachment, cognitive enhancement, and alertness than one of its main metabolites, Amphetamine.

**Figure 1:**
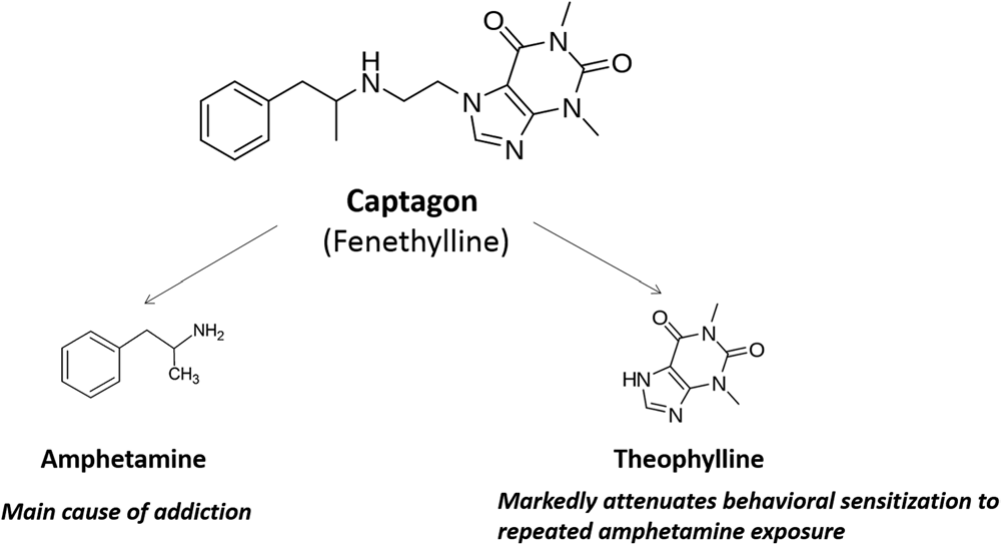
Metabolic scheme of Captagon (Fenethylline). Captagon when orally dosed will go through the oxidative metabolism via cytochrome P450 (CYP450) enzyme, and in final be metabolized into 24.5% Amphetamine and 13.7% Theophylline. Amphetamine is the main cause of addiction, while Theophylline reduces the behavioral sensitization to repeated Amphetamine exposure, thus explain the less addictiveness of Fenethylline than Amphetamine.

Captagon is metabolized into Amphetamine (24.5% of oral dose) and Theophylline (13.7% of oral dose), as shown in Fig. 1. Thus, the pharmacological effect of Captagon is considered the result of the combined action of these two metabolites^3,6^. Amphetamine, the main metabolite of Captagon, is a CNS stimulator that can increase alertness, boost concentration/physical performance, and provide a feeling of well-being, confidence, and aggression. It can be used for the treatment of obesity, narcolepsy, and attention deficit hyperactive disorder (ADHD)^7^. Amphetamine is reportedly an agonist on central 5-HT receptors^8–10^ and may inhibit monoamine oxidase (MAO)^11^, possibly causing hallucinations, violent behavior, loss of appetite, and more. Theophylline, another metabolite of Captagon, is a weak stimulator resembling caffeine^12^. Theophylline has a narrow therapeutic window due to its unwanted side effects including cardia dysrhythmia seizures, gastrointestinal disturbances, and drug-drug interactions^13^. Captagon was considered a co-drug (or mutual prodrug) of Amphetamine and Theophylline. However, the effects of Fenethylline is different from those of Amphetamine qualitatively and quantitatively. First, previous literature^6,14^ reported that Captagon is more lipophilic than both Theophylline and Amphetamine, resulting in easier absorption into the CNS and a faster stimulating effect than either drug^2^. Second, according to a recent report by Wenthur and co-workers^2^, “the penetration of Amphetamine into brain tissue following release from Fenethylline lags behind that of directly administered Amphetamine” which may explain why Captagon is less addictive than Amphetamine, i.e. because Fenethylline-sourced Amphetamine is less likely to accumulate in the brain. Third, unlike vasoconstrictors such as Amphetamines, Captagon does not increase the patient’s blood pressure because Theophylline possesses vasodilating properties, allowing its use by those with cardiovascular disorders^6^. More available information regarding Captagon/Fenethylline can be found in a recent minireview paper published by Katselou and co-workers^6^.

Captagon is an addictive drug that fuels conflicts in the Middle East^4^. Fighters on Captagon or Amphetamines may feel a sense of well-being, euphoria and invincibility. It is clear that these drugs are favored to suppress pain and increase aggression in soldiers. The use of these drugs is not limited to soldiers but also to the civilian population in areas of prevailing hopelessness or helplessness. Due to its great profitability, many Captagon seizure cases occur in the Middle East and Europe according to the United Nations Office on Drugs and Crime (UNODC). For example, Al-Imam and co-workers wrote in their article, “11,000 Captagon tablets were seized in the city of Al-Nasiryia, a city to the southeast of the Iraqi capital Baghdad.” And they also mentioned in their article that “illegal Captagon was seized by fighters of the Islamic State in Iraq and other militant groups in Syria (more than 1.4 million tablets), in Turky (107.5 kilograms of drug) and in Egypt (8.8 million pills)”^15^. Furthermore, in 2013 Dubai authorities seized 4.6 million Captagon pills. In May 2017, French authorities reported confiscating about 300 pounds of Captagon, seizing 750,000 pills worth an estimated $1.7 million.

Although Captagon was first synthesized in 1961, its underlying addictive mechanism, potential drug-drug interactions with Amphetamine and Theophylline, side effects such as hallucination, and detailed interactions between Captagon/Amphetamine/Theophylline and their targets have been relatively ignored by academic research and industrial studies for over 56 years.

To address the above questions, we performed computational systems pharmacology analyses for Captagon and its metabolites, Amphetamine and Theophylline. First, we analyzed the signaling pathways for both Amphetamine and Theophylline. We found that Amphetamine is the main cause for Captagon addiction, and Theophylline can block adenosine receptors in the brain, attenuating Amphetamine’s behavior. Subsequently, we docked Amphetamine/Theophylline into their reported targets to explore their detailed interactions at a molecular level. Finally, to study the side effects of Amphetamine and Captagon, we carried out off-target predictions using our established hallucinogen-related chemogenomics knowledgebase and in-house computational chemogenomics tools. Overall, such drug-drug combinations could potentially be used to treat drug abuse and addiction. Our studies provided a detailed insight into the addiction mechanism of Captagon and its metabolites.

## Materials and Methods

### Homology Modeling of TAAR1 and Adenosine A2b Receptor

In this study, we built two GPCR models including trace amine-associated receptor 1 (TAAR1) and adenosine A2b receptor (A2bR). First, we used a β1AR crystal structure (PDB ID: 2Y00, Resolution: 2.5 Å)^16^ as the template to build the homology model of TAAR1 and found the sequence identity between the two proteins is about 35%. Then, a homology model of the adenosine A2b receptor was built using the crystal structure of the adenosine A2a receptor (PDB ID: 3RFM, Resolution: 3.6 Å)^17^ as the template, finding the sequence identity between the two proteins to be about 66%. These crystal structures were downloaded from the Protein Data Bank (http://www.pdb.org/pdb/). SYBYL-X 1.3^18^ was used to repair all residues and minimize energy.

The full sequences of human TAAR1 receptor (TAAR1_HUMAN, 339 residues) and adenosine A2b receptor (AA2BR_HUMAN or P29275, 332 residues) were retrieved from UniProtKB/Swiss-Prot (http://www.uniprot.org/uniprot/). We then truncated the residues according to their templates for sequence alignment and homology modeling using our reported protocol^19^. The disulfide bridge(s) was also patched for each homology model.

Modeller 9.18^20^ was used to construct these homology models. Once the 3D models were generated, SYBYL-X 1.3 was used to perform the energy minimizations. Briefly, the parameters defined in SYBYL are: Gradient set to 0.5 kcal/mol, Max iterations set to 5000, force field set to MMFF94, and charge method set to MMFF94. All of these settings are the same as in our previous works^21–25^. Then, proSA-web Z-scores^26^ and PROCHECK Ramachandran plots^27^ were used to validate the models.

### Docking Study of Ligand-Receptors

We used the MOLCAD module implemented in SYBYL-X 1.3 to explore the potential binding pocket for GPCR receptors. The docking program Surflex-Dock GeomX (SFXC) in SYBYL-X 1.3 was applied to construct receptor-ligand complexes in which the docking scores were expressed in −log_10_ (K_d_)^28^. The main protocols or parameters of docking were addressed in our previous publications^21–25^. Briefly, the docking parameters used were: (a) number of starting conformations per ligand set to 10, max conformations per fragment set to 20; (b) maximum number of rotatable bonds per molecule set to 100; (c) flags were turned on at pre-dock minimization, post-dock minimization, molecule fragmentation, and soft grid treatment; (d) activate spin alignment method with density of search set to 9.0; and (e) number of spins per alignment set to 12.

### Hallucinogen-Specific Chemogenomics Knowledgebase and Systems Pharmacology Analysis

We have constructed a Hallucinogen-Specific Chemogenomics Database^29^ that can be used for target, off-target, or additional identification and network systems pharmacology analysis of small molecules and their potential targets. Several in-house chemoinformatics tools were also used, including TargetHunter, HTDocking, Blood-Brain Barrier (BBB) Predictor, and more^30,31^. HallucinogenPlatform (http://www.cbligand.org/hallucinogen/) collected 144 hallucinogen-related target proteins and 145 chemical compounds associated with these targets in 6,721 assays and 23,598 references.

In our work, we applied our HallucinogenPlatform and established chemoinformatics tools such as HTDocking to perform network systems pharmacological analysis on Amphetamine and Captagon. First, Amphetamine and Captagon were docked into the target proteins’ pockets. We then matched these predicted target proteins to Amphetamine and Captagon according to their docking scores. Targets with higher docking scores may have higher binding affinities and therefore a greater chance of interacting with Amphetamine/Captagon. Next, we mapped out a pharmacological network of interactions between drug compounds and target proteins at the molecular level^32,33^. Cytoscape 3.4.0^34^ was used to generate, analyze and visualize the network of targets and drugs/compounds as described previously^33^.

### Molecular Dynamics (MD) Simulation

Two systems were set up for molecular dynamics simulations using the web-based tool CHARMM-GUI^35,36^. For the TAAR1 receptor complexed with Amphetamine, the system included 256 1-palmitoyl-2-oleoyl-sn-glycero-3-phosphocholine (POPC) lipids, 22550 water molecules, and 60 Na^+^ ions and 69 Cl^−^ ions. For the A2aR receptor complexed with Theophylline, the system included 254 POPC lipids, 22427 water molecules, 60 Na^+^ ions, and 67 Cl^−^ ions. The initial configurations of protein receptors and ligands were taken from docking studies. The sizes of the initial simulation boxes were ~100 Å * 100 Å * 117 Å.

The AMBER ff14SB force field^37^ was applied to proteins and the AMBER Lipid14 force field^38^ was applied to lipids. Water molecules were treated with the TIP3P water model^39^. The partial atomic charges of ligands were derived by the restrained electrostatic potential (RESP) method^40^ to fit the HF/6–31 G* electrostatic potentials generated using the GAUSSIAN 16 software package^41^. Other force field parameters came from GAFF in AMBER16^42^. Residue topologies for ligands were prepared using the ANTECHAMBER module^43^.

The MD simulations were carried out using the PMEMD.mpi and PMEMD.cuda modules in the AMBER16^44–46^ package. First, several minimization steps were carried out for the systems to avoid possible steric crashes. Then, each system was gradually heated from 0 K to 300 K during the heating stage and kept at 300 K during the following equilibrium and production stages. A time step of 1 fs was used for the heating and first part of the equilibrium stage, and 2 fs was used for the remaining part of the equilibrium stage and the entire production stage. A periodic boundary condition was employed to maintain constant temperature and pressure (NPT) ensembles. Pressure was set at 1 atm and controlled by the anisotropic (x_−_, y_−_, z_−_) pressure scaling protocol applied in AMBER with a pressure relaxation time of 1 ps. Temperature was regulated using Langevin dynamics with a collision frequency of 2 ps^−1^ ^47,48^. The Particle Mesh Ewald (PME) method^49,50^ was adopted to handle long-range electrostatics and a 10 Å cutoff was set to treat real-space interactions. All covalent bonds involving hydrogen atoms were constrained with the SHAKE algorithm^51^. The simulations time for the production stage for each system was 200 ns and the coordinates of simulated systems were saved every 100 ps.

For the saved trajectories of MD simulations, Molecular Mechanics/Generalized Born Surface Area (MM/GBSA) binding free energies were calculated^52,53^ and free energy decompositions were performed. The interaction energies between each residue and ligand were extracted.

## Results

### Signaling Pathway Analysis for Amphetamine, a Stimulant with Addiction

Amphetamine, a dopamine inducer, has been reported to not only provide a sense of euphoria but also to contribute to addiction. Addiction to Amphetamine mainly arises from the increased release of dopamine in “the mesoaccumbens dopamine pathway, extending from the ventral tegmental area (VTA) of the midbrain to the nucleus accumbens (NAc)”^54^. NAc, also known as the ventral striatum (VS), has been identified to be “the critical shared substrate for the reinforcing effect of Amphetamines, cocaine, and other addictive drugs”^55^.

**Figure 2:**
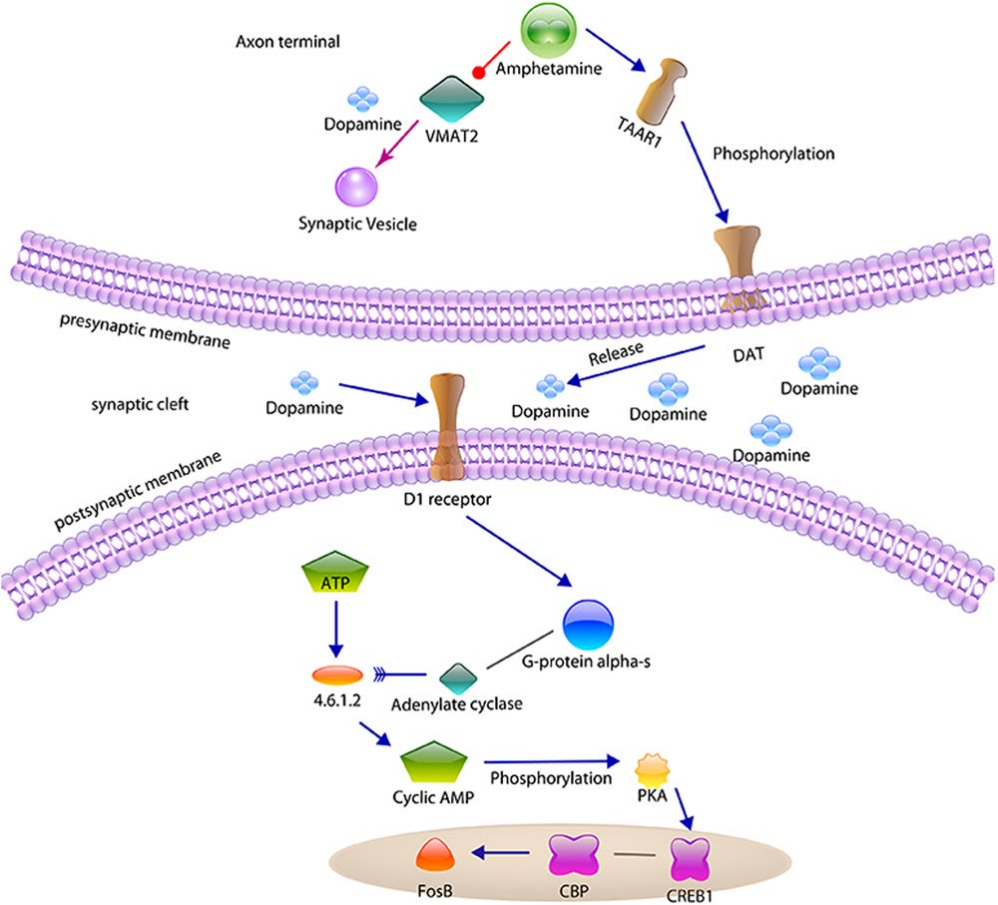
Signaling pathway analysis for Amphetamine, a stimulant with addiction. On one way, Amphetamine inhibits the VMAT2 to transport dopamine into synaptic vesicles. On the other, Amphetamine binds with TAAR1 and then increase the phosphorylation of DAT, which release more dopamine into the synaptic cleft. D1 receptor on the postsynaptic membrane and the following signaling transduction are activated to lead to the addiction factor FosB accumulation.

Amphetamine is reported to be an inhibitor of vesicular monoamine transporter 2 (VMAT2)^56^, but an agonist of Trace amine-associated receptor 1 (TAAR1)^57^. As shown in Fig. 2, VMAT2^58^ trans-membrane transporter mainly delivers neurotransmitters from the cellular cytosol into synaptic vesicles^59^. TAAR1, a class A GPCR^57,60^, is primarily located in peripheral tissues^61^, glial cells^62^, and neurons^63^ and is responsible for regulating neurotransmission in dopamine, norepinephrine, and serotonin neurons in the CNS^64^. On one hand, the binding of Amphetamine to VMAT2 increases the release of dopamine from vesicles^63,65^. On the other hand, TAAR1 can reverse the dopamine transporter (DAT) function by activating protein kinase A (PKA) and protein kinase C (PKC), allowing DAT to transport dopamine into the intracellular region and therefore the synaptic cleft^63,66^. This activates the D1 dopamine receptor (D1R) on the post-synaptic neuron. D1R, a D1-like GPCR, can bind to G-proteins and increase the cAMP level, phosphorylating cAMP-response element binding protein 1 (CREB1) and increasing ΔFosB levels^67,68^. ΔFosB, a master control protein, governs the activity of several other transcriptional (e.g. AP-1) and epigenetic regulatory proteins and represses the c-Fos gene. c-Fos repression acts as a molecular switch that enables the accumulation of ΔFosB in the neuron. As a result, ∆FosB will persistently overexpress and accumulate in the D1-type medium spiny neurons that exist in the nucleus accumbens following repeated high-dose exposures to Amphetamines, causing the addiction.

### Signaling Pathway Analysis for Theophylline, an Antagonist of Adenosine A2a Receptor for Drug Abuse

Theophylline, a weak stimulator chemically and pharmacologically resembling caffeine^6^, can inhibit phosphodiesterase (causing an increase in cAMP levels) and block adenosine receptors.

**Figure 3:**
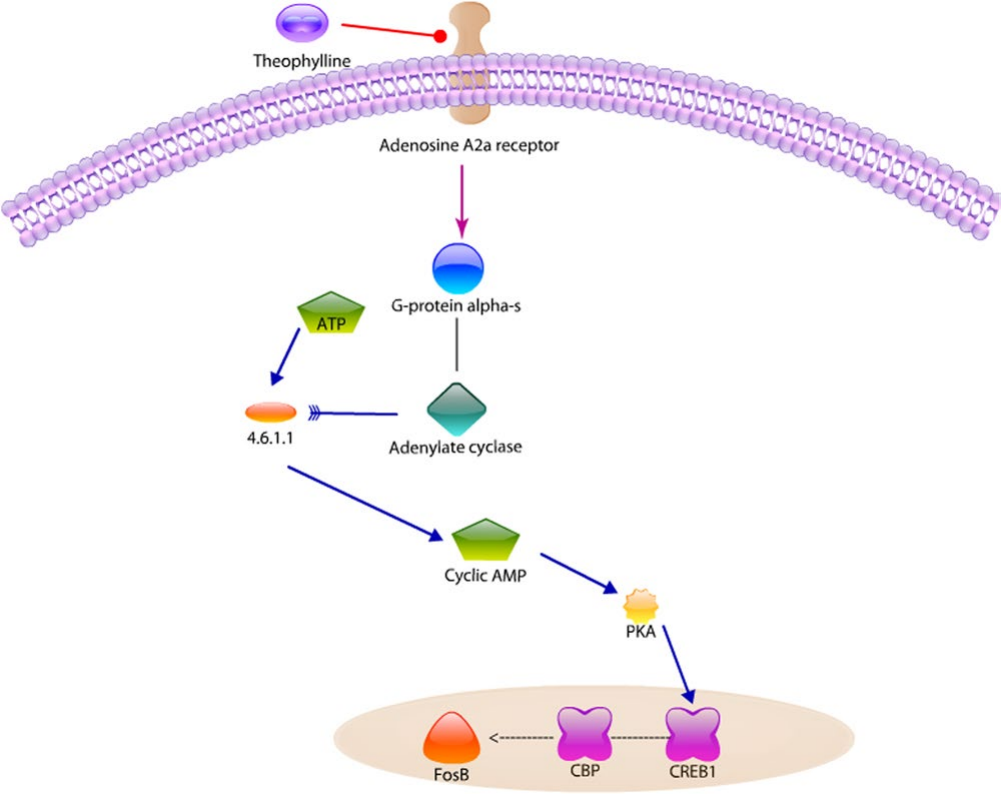
Signaling pathway analysis for Theophylline, an antagonist of adenosine A2a receptor for drug abuse. The inhibition of adenosine A2a receptor by theophylline can reduce the expression of CREB1. We hypothesized that the crosstalk of the pathway between Amphetamine and Theophylline, that is, the reduced expression of CREB1 decreasing the FosB accumulation, will help explain the experimental results that Theophylline reduces the behavioral sensitization to repeated amphetamine exposure.

As shown in Fig. 3, A2aR is a GPCR whose preferred endogenous agonist is adenosine^69^. A2aR is an attractive therapeutic target for cardiac function^70^, insomnia^71^, inflammatory response^72^, pain^71^, Parkinson’s disease^70,73^, and drug addiction^71,74–77^. We found that blockage of A2aR by Theophylline inhibits CREB1 phosphorylation. Some literature^75,76^ has reported that “the inactivation/antagonists of adenosine A2aR selectively attenuates Amphetamine-induced behavioral sensitization,” implying that blockage of A2aR by Theophylline will attenuate the addiction of Amphetamine and Captagon. Jiang-Fan Chen *et al*. administrated the same dose Amphetamine in both WT and A2aR KO mice for 7 days, and found that the enhancement of significant locomotion induced by amphetamine was shown in the WT rats, in contrast, not in the A2aR KO mice^75^. Elena Bastia *et al*. used A2aR knockout (A2aR KO) mice model, showing that pairing daily amphetamine doses with their test A2aR antagonist (SCH58261 or KW-6002) prevented locomotor sensitization on day 8 in mice^76^. Although no direct pathway showed that Theophylline affects the pathway of Amphetamine addiction, it may affect the accumulation of ∆FosB (dash line in Fig. 3, our hypothesis).

### Interactions of Amphetamine with TAAR1

Amphetamine directly targets TAAR1 as an agonist. We explored in detail the interactions between TAAR1 and Amphetamine. No TAAR1 crystal structure has been reported, so we built the homology model using β1AR as a template (PDB ID: 2Y00, Resolution: 2.5 Å)^16^, with a sequence identity of about 35%. Then the model was further validated by energy minimization, proSA-web Z-scores^26^, and PROCHECK Ramachandran plots^27^.

**Figure 4:**
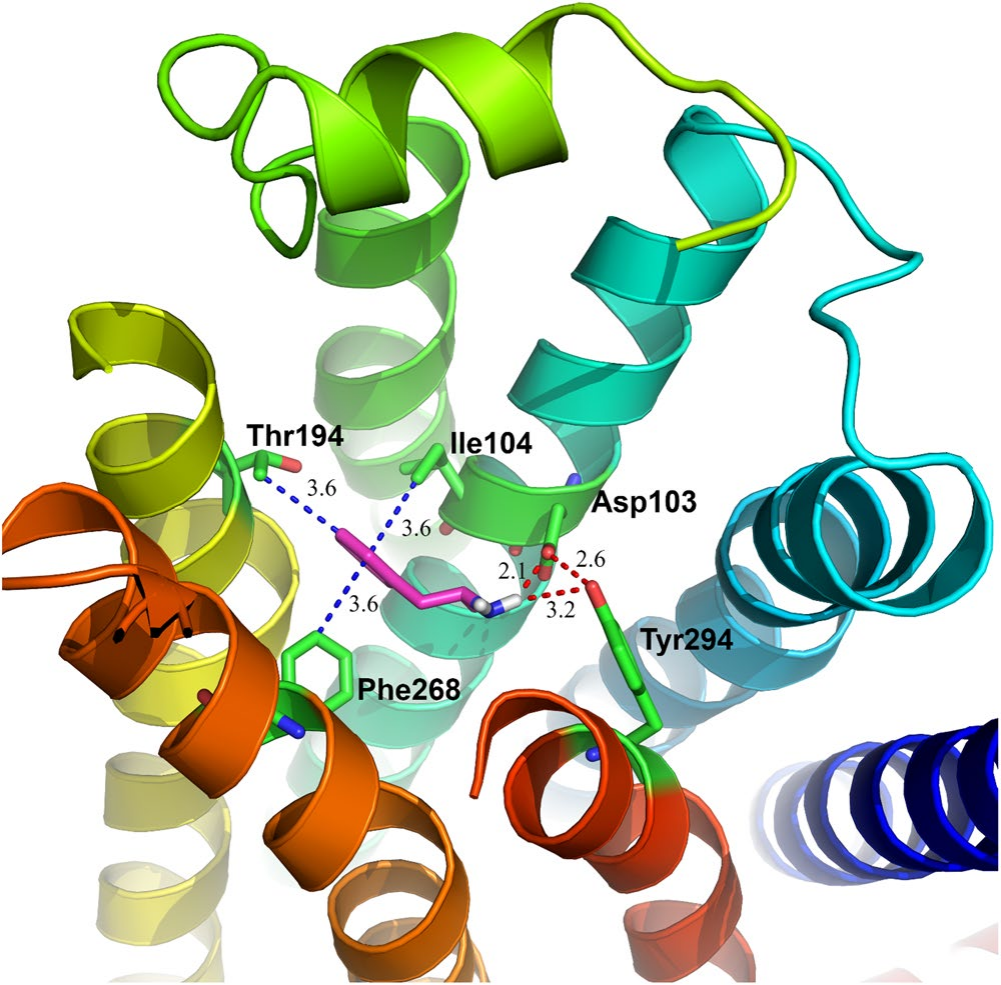
Detailed interactions between Amphetamine and TAAR1. The key residues on the binding pocket of TAAR1 involves Asp103, Tyr294, Ile104, Thr194 and Phe268. Hydrogen bonding interaction will be formed between the nitrogen atom belonging to Amphetamine and Asp103 (~2.1 Å) and Tyr294 (~3.2 Å), the other hydrogen bond is between Asp103 and Tyr294. While Ile104 (~3.6 Å), Thr194 (~3.6 Å) and Phe268 (~3.6 Å) contributed to the hydrophobic interactions with benzene ring of Amphetamine.

As shown in Fig. 4, our results showed that the potential binding pocket in TAAR1 is formed by TM3, TM5, TM6 and TM7. Several important residues formed strong hydrophobic interactions with Amphetamine, including Ile104(3.33) in TM3, Thr194(5.43) in TM5, and Phe268(6.52) in TM6, with a bond distance of 3.6 Å. Asp103(3.32) in TM3 and Tyr294(7.42) in TM7 formed strong hydrogen bonds with Amphetamine, with distances of about 2.1 Å and 3.2 Å, respectively. Interestingly, we found an additional hydrogen bond between Asp103 and Tyr294, making the previous two hydrogen bonds more stable. All of these residues in our work are supported by modeling data reported by Tan *et al*.^78^ and docking data calculated by Cichero *et al*.^79^. For further validation of the binding mode, we also carried out a molecular dynamics (MD) simulation as described below.

### Interactions of Theophylline with A2aR and A2bR

Using the same protocol, we studied interactions between A2aR (and A2bR) and Theophylline at a molecular level. The crystal structure of A2aR (PDB ID: 3RFM, Resolution: 3.6 Å)^17^ was used to construct the homology model of A2bR, with a sequence identity of about 66%.

We first docked the reported compound caffeine back to the crystal structure of A2aR as a validation of our docking protocol as shown in Supporting Figure S1a. The binding pocket of A2aR is formed by Helices I, II, III, V, VI, and VII, and surrounded by important residues including Asn253 (hydrophilic), His278 (hydrophilic), Val84 (hydrophobic), Phe168 (hydrophobic), Met177 (hydrophobic), Leu249 (hydrophobic), Met270 (hydrophobic, not shown), and Ile274(hydrophobic). We found that Asn253 formed strong hydrogen bonding (3.3 Å) with caffeine, indicating this residue is very important for ligand recognition. Moreover, six other residues including Val84 (3.8 Å), Phe168 (3.6 Å), Met177 (3.3 Å), Leu249 (3.8 Å), Met270 (3.9 Å, not shown), and Ile274 (4.1 Å) formed strong hydrophobic interactions with caffeine. Moreover, our docking results showed that the docked caffeine (yellow sticks) overlapped well with the crystallized compound (cyan) with a root mean square deviation (RMSD) of 0.3 Å, indicating our docking protocol is reliable.

**Figure 5:**
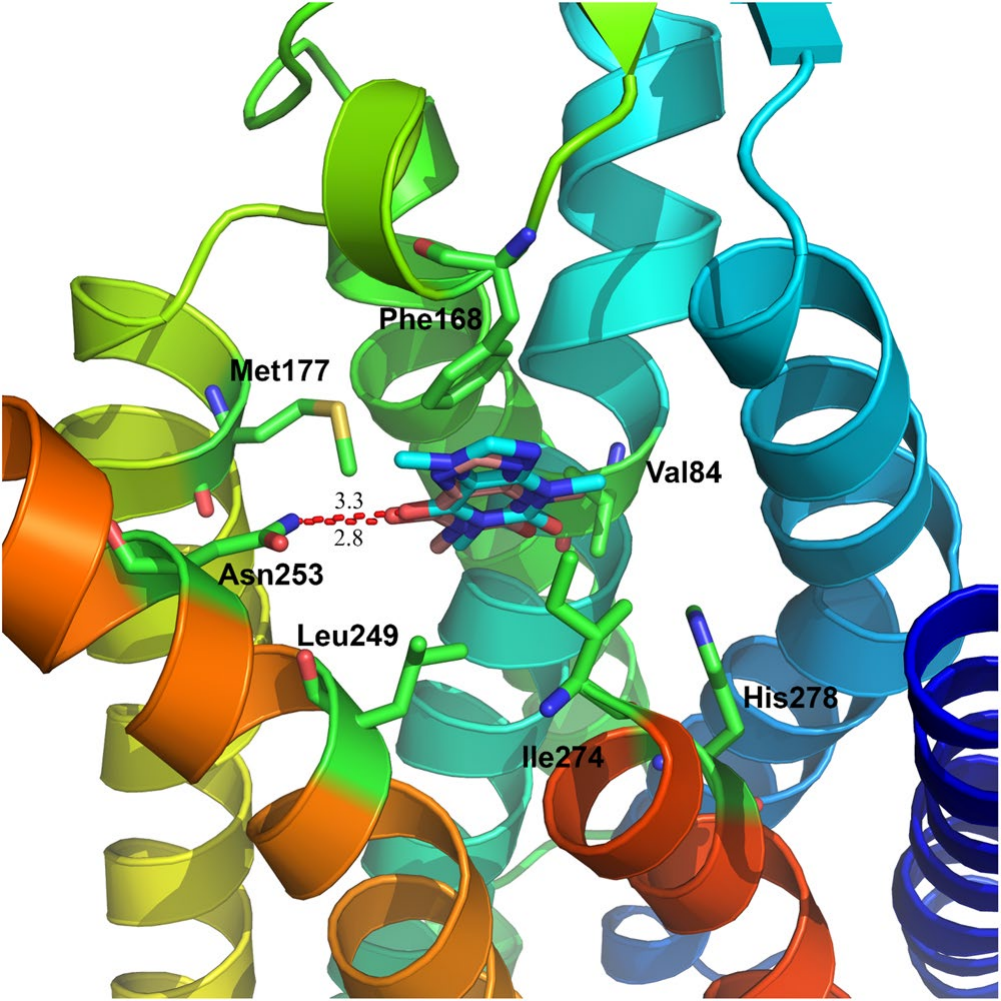
Detailed interactions between Theophylline and A2aR. The key residues on the binding pocket of A2aR involved Asn253, Val84, Phe168, Leu249, Ile274, Met177, and His278. Our docking data showed that Theophylline (salmon) overlapped with caffeine (cyan) very well, which indicates the reliability of our docking methods. Moreover, these two compounds share almost the same interactions. Briefly, Asn253 on both caffeine and Theophylline has the hydrophilic bond with Amphetamine in 3.3 Å and 2.8 Å, while Val84, Phe168 and Leu249 formed strong hydrophobic interactions with Theophylline.

We then docked Theophylline into A2aR using the same protocol and compared the binding mode of Theophylline (salmon) with that of caffeine (cyan) with their co-crystal structures, as shown in Fig. 5. Our docking data showed that Theophylline (salmon) overlapped with caffeine (cyan) very well, with an RMSD of 0.15 Å. Moreover, these two compounds shared almost the same interactions. Briefly, Asn253 (2.8 Å) formed hydrogen bonding interactions with Theophylline (salmon), while Val84, Phe168, Met177, Leu249, Met270, and Ile274 interacted with Theophylline through strong hydrophobic interactions.

For comparison, we also docked caffeine and Theophylline into A2bR as shown in Supporting Figure S1b. Our docking results showed that both caffeine and Theophylline shared similar binding modes and interactions with these two receptors.

### Molecular Dynamics (MD) Simulation of Amphetamine and Theophylline with Target Receptors

To study the dynamics and mechanisms of protein-ligand binding between Amphetamine, Theophylline, and related receptors, we carried out 200 ns-MD simulations for the TAAR1-Amphetamine complex and the A2aR-Theophylline system.

**Figure 6:**
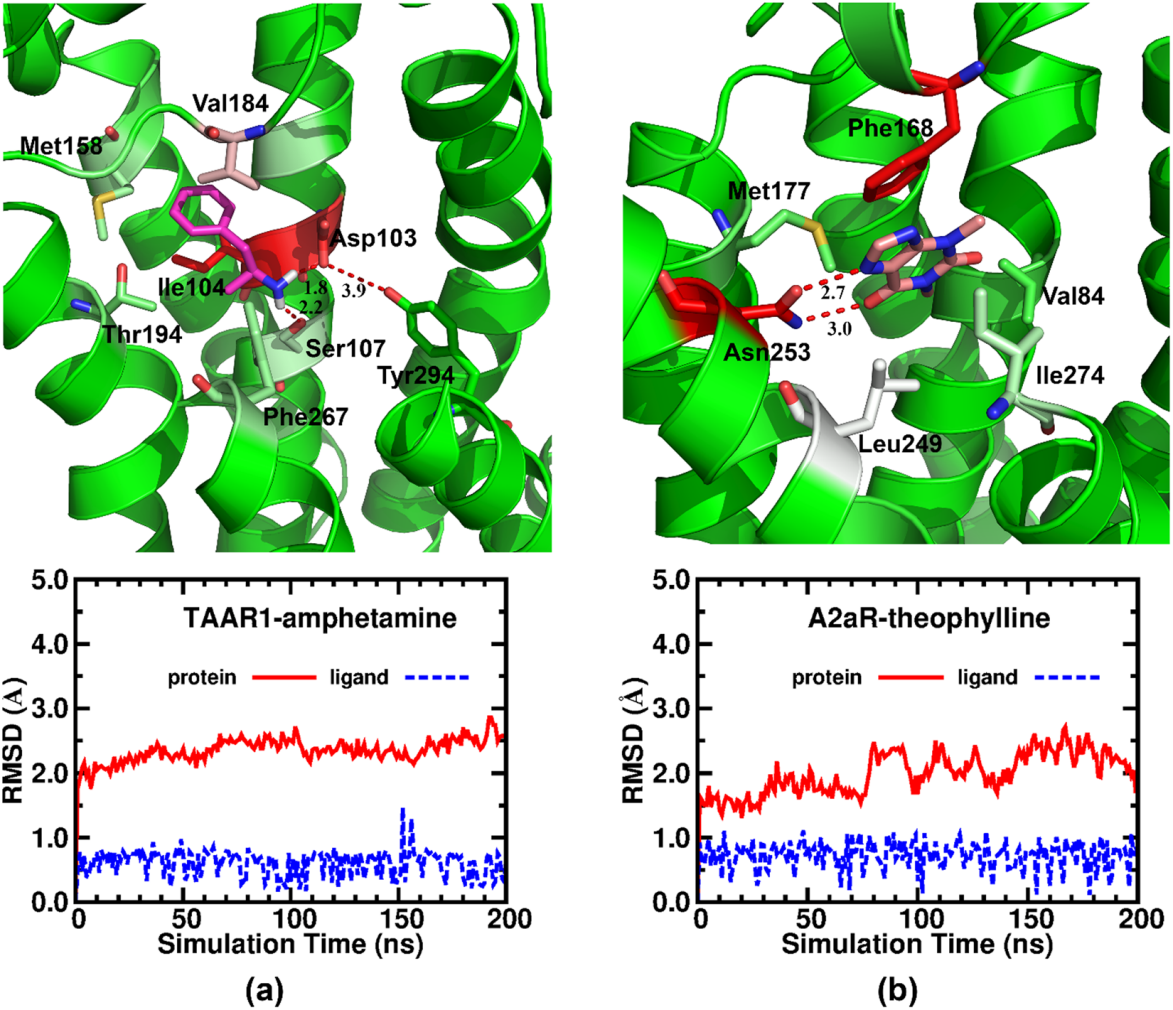
200 ns MD simulation for Amphetamine and Theophylline. (**a**) MD simulation and binding mode of Amphetamine with TAAR1. TAAR1-amphetamine system is stable within 200 ns simulation process and an additional hydrogen bond was formed between Amphetamine and Ser107 (2.2 Å). Amphetamine also interacted with Val184 in the ECL2 (The Extracellular Loop 2) domain and Met158 in TM4 by hydrophobic interactions. (**b**) MD simulation and binding mode of Theophylline within A2aR. A2aR-theophylline system is stable within 200 ns simulation process but shown a small rotation because of the hydrogen bond between Theophylline and A2aR.

Detailed interactions after the MD simulations and the RMSDs of the receptors and ligands compared to their initial configurations were plotted in Fig. 6. Our MD results showed that the RMSDs of both protein receptors were small (around 2.5 Å) during 200 ns simulations, indicating the systems were stable and reasonable. The RMSDs of both ligands during the MD simulations were more stable than that of the receptors (less than 1 Å), demonstrating that our docking results and poses of the ligands were reliable. Next, Fig. 6a shows the detailed interactions between Amphetamine and TAAR1. The data showed that the hydrogen bond between Amphetamine and Asp103 was stable (1.8 Å). Importantly, an additional hydrogen bond was formed between Amphetamine and Ser107 (2.2 Å). Moreover, Amphetamine interacted with Val184 in the ECL2 (The Extracellular Loop 2) domain and Met158 in TM4, forming strong hydrophobic interactions. Finally, Fig. 6b shows that Theophylline had a slight rotation during the MD simulation, forming two strong hydrogen bonds with Asn253 (2.7 Å/3.0 Å). The hydrophobic interactions kept stable during the simulations.

We further characterized the protein-ligand binding by calculating the interaction energy (IE) between the ligand and each residue of the receptor using MM-GB/SA free energy decomposition analysis, as shown in Table 1. We found that the MD results correlated very well with our previously described docking studies. For example, Asp103 (−5.46 kcal/mol, red in Fig. 6a), Ile104 (−4.19 kcal/mol, red in Fig. 6a), and Val184 (−5.46 kcal/mol, light pink in Fig. 6a) in TAAR1 contributed greatly to the binding of Amphetamine, while Asn253 (−6.24 kcal/mol, red in Fig. 6b), Phe168 (−6.02 kcal/mol, red in Fig. 6b), and Leu249 (−3.00 kcal/mol, white in Fig. 6b) in A2aR played important roles in the recognition of Theophylline.

**Table 1:** Key residues of TAAR1 binding to Amphetamine and A2aR binding to Theophylline. IE (interaction energy) was calculated by MM-GB/SA (kcal/mol).

Receptor	Residue	IE
TAAR1	Asp103	−5.46
	Ile104	−4.19
	Ser107	−1.60
	Met158	−1.28
	Val184	−2.56
	Thr194	−1.07
	Phe267	−1.32
	Phe268	−0.57
A2aR	Val84	−0.93
	Phe168	−6.02
	Met177	−1.28
	Leu249	−3.00
	Asn253	−6.24
	Met270	−1.35
	Ile274	−2.31

## Discussion

### Drug-Drug Interactions between Amphetamine and Theophylline

Some literature reported that Theophylline is the substrate of CYP2D6^80,81^, an enzyme involved in many metabolic processes. Moreover, Amphetamine is reported to be a substrate and inhibitor of CYP2D6^81,82^. As a result, Theophylline will be eliminated more slowly due to competition and/or blockage of CYP2D6 by Amphetamine. Thus, some side effects of Theophylline like nausea, diarrhea, and an increase in heart rate may last longer if its plasma concentration is high enough. Because Theophylline only constitutes 13.7% of the oral dose of Captagon, we suggest that the risk of overdose due to Theophylline is low. Importantly, blockage of A2aR by Theophylline should last longer due to this prolonged elimination time. In turn, the effects of Theophylline on decreasing Amphetamine addiction will last longer than in a scenario without this competitive elimination.

### Knowledgebase-guided Off-target Predictions for Amphetamine/Captagon and Associated Side Effects

Previously, we constructed a Hallucinogen-Specific Chemogenomics Knowledgebase^29^ that can be used for target, off target, or additional identification and network systems pharmacology analysis of small molecules and their potential targets. In our current work, we adapted a small but specific dataset with twelve 5-HT receptors in our knowledgebase to explore the side effects (e.g. hallucinations) for both Amphetamine and Captagon. All of these 5-HT or serotonin receptors are GPCRs widely distributed throughout the CNS, reportedly associated with depression, anxiety, and drug addiction^83^. Here, we docked both Amphetamine and Captagon into 5-HT receptors to predict potential off-targets.

**Figure 7:**
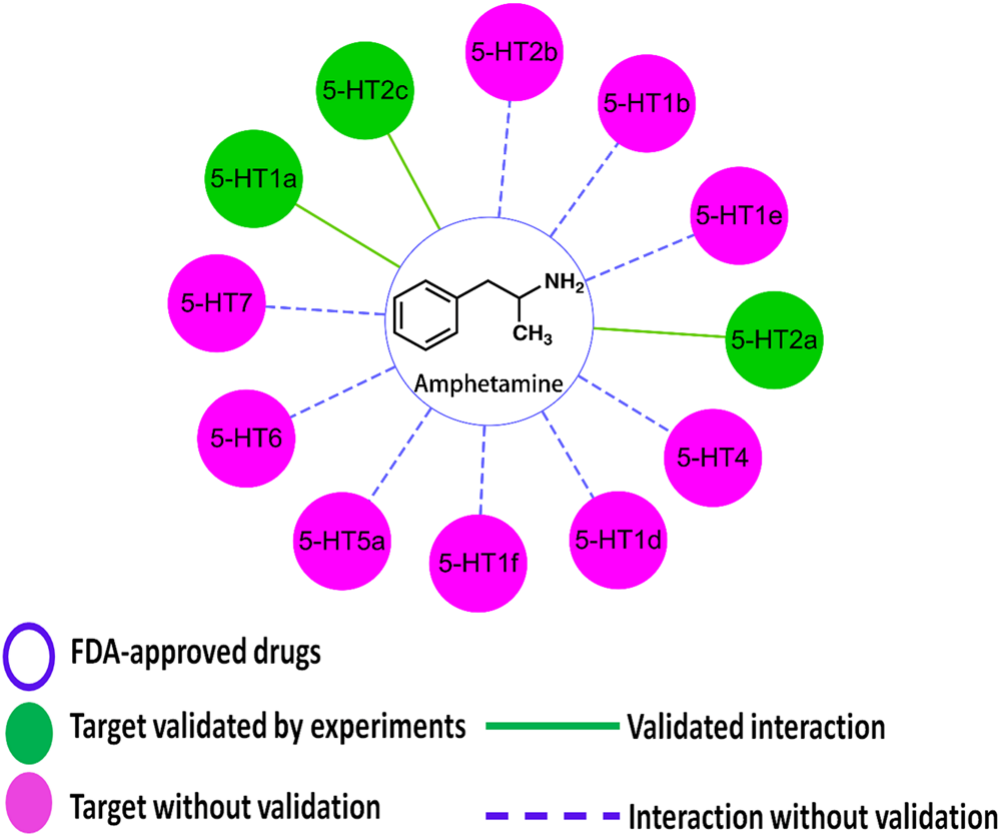
Network systems pharmacology analysis of Amphetamine and the predicted targets. Serotonin or 5-hydroxytryptamine (5-HT) receptor family has been linked to the regulation of mood and a series of physical behaviors like hallucination and reward. We predicted Amphetamine can also target at this kind of receptors (shown in purple) with different affinity using our established knowledgebase and HTDocking target identification program. Plus, we found there has been experimental affinity data about 5-HT1a, 5-HT2a and 5-HT2c (shown in green).

As shown in Fig. 7, additional potential Amphetamine target proteins were predicted and ranked by their docking scores (shown in Table 2) in the form of an interaction network (green one-known target, purple one-predicted target). Our results showed that most of the docking scores were not high due to the huge size of the binding pocket in the receptor and the small size of the Amphetamine molecule. However, our predicted results correlated well with the known therapeutic targets of Amphetamine. For example, the docking scores of Amphetamines with 5-HT2a and 5-HT2c were 4.97 and 5.14, which is well correlated with their experimental data. The docking score of Amphetamine with 5-HT1a is 5.12 with experimental data of 7.66 µM. Importantly, we predicted two potential targets for Amphetamine, including 5-HT1f (docking score of 5.26) and 5-HT1e (docking score of 6.14), as shown in Table 2.

**Table 2:** The predicted protein targets for Amphetamine/Captagon using HTDocking program (docking score represents −logKd).

Protein	Score (Amphetamine)	Score (Fenethylline)	Experimental data of Amphetamine	Gene name
5-HT1a	5.21	7.56	7.66 µM	HTR1A
5-HT1b	4.81	7.74		HTR1B
5-HT1d	4.16	8.20		HTR1D
5-HT1e	6.14	7.22		HTR1E
5-HT1f	5.26	7.69		HTR1F
5-HT2a	4.97	8.81	>10 µM	HTR2A
5-HT2b	5.00	6.84		HTR2B
5-HT2c	5.14	8.84	>10 µM	HTR2C
5-HT4	4.90	8.75		HTR4
5-HT5a	5.04	8.91		HTR5A
5-HT6	4.40	8.31		HTR6
5-HT7	4.72	9.93		HTR7

**Figure 8:**
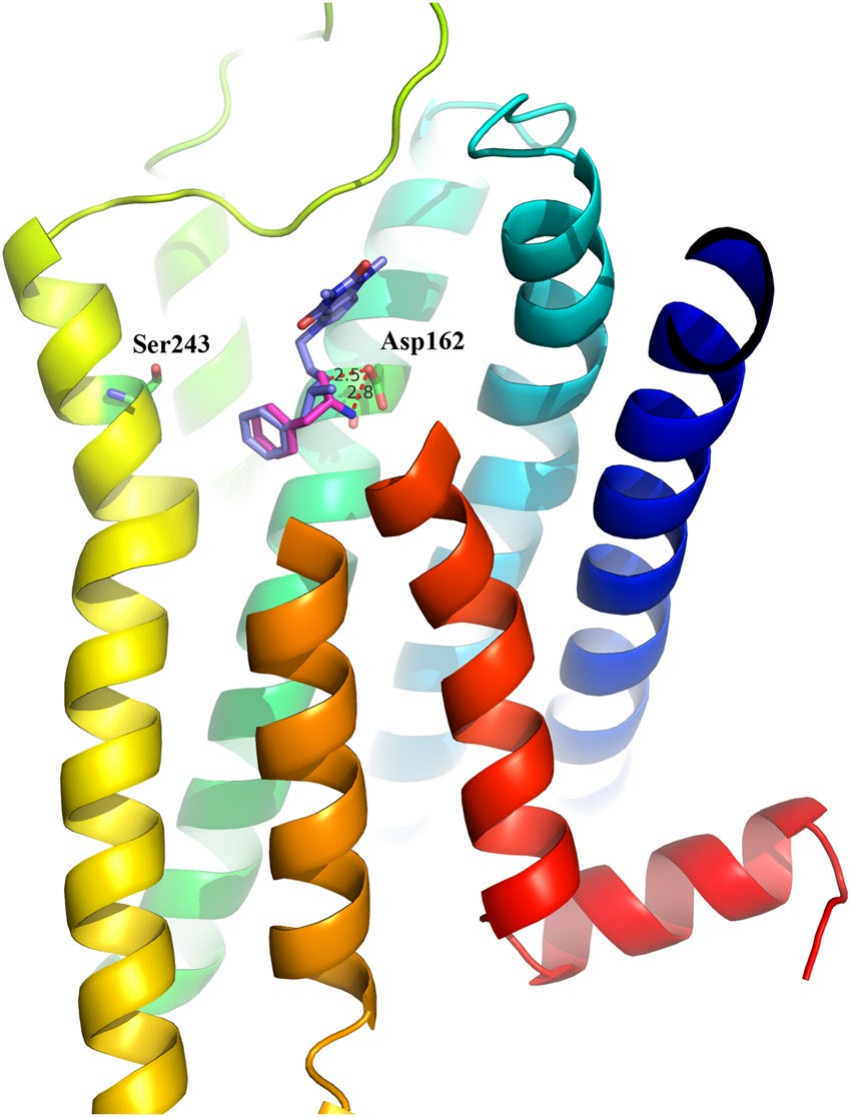
Predicted binding mode of Amphetamine (purple) and Captagon (slate) within 5HT7. Our docking result showed that Amphetamine and Captagon overlapped very well and that Asp162 (D3.32) formed strong hydrogen bonds with both Amphetamine (~2.8 Å) and Captagon (~2.8 Å). For further validation, we will carry out future experiments.

In terms of Captagon lipophilicity^6,14^, we also calculated the docking scores of Captagon before metabolism within these 5-HT receptors, as shown in Table 2. All the docking scores of target proteins for Captagon were higher than those of Amphetamine, due to the latter’s more flexible structure and stronger interactions with the receptor. In our present work, we showed the detailed interaction of Captagon with its most probable target, 5-HT7. For comparison, we aligned the predicted binding mode of Amphetamine (purple) and Captagon (slate) with 5-HT7, as shown in Fig. 8. Our docking data showed that Amphetamine and Captagon overlapped very well and that Asp162 (D3.32) formed strong hydrogen bonds with ligands. For further validation, we will carry out future experiments.

### Insight into Captagon

The data analyses are summarized: (1) Captagon when orally dosed will be metabolized into 24.5% Amphetamine and 13.7% Theophylline; (2) Captagon is more lipophilic than both Theophylline and Amphetamine, resulting in easier absorption into the CNS; (3) Amphetamine, a TAAR1 agonist that enhances dopamine signaling (causing increased irritability, aggression, etc.), is the main cause of Captagon addiction; (4) Theophylline, an A2aR antagonist that blocks adenosine receptors in the brain (causing restlessness and painlessness), may attenuate the behavioral sensitization caused by Amphetamine; (5) the drug-drug interactions between Amphetamine and Theophylline slow the metabolism and elimination of Theophylline through competition and/or blockage of CYP2D6 by Amphetamine; and, (6) Theophylline and Amphetamine act synergistically to augment Captagon’s psychoactive effects beyond those caused by Amphetamines alone.

Moreover, A2aR reportedly has antagonistic interactions with the D2 receptor through heterodimer formation^84^. Poleszak E et al. reported that Adenosine A2a receptor antagonist, DMPX (3 and 6 mg/kg ip) can attenuate the amphetamine-induced stereotypy in male Wistar rats^85,86^. This complex exists in neurons found in the nucleus accumbens and ventral and dorsal striatopallidal GABAergic neurons, reportedly the main areas involved in addiction^87^. Theophylline, a known A2aR antagonist specifically targets the A2aR protomer, increasing the affinity of dopamine for the D2 receptor and facilitating D2-mediated Gi/o signaling. D2 neurons are associated with suppressing addictive drug rewards, opposite to D1-expressing neurons reinforcing rewards^88^. Thus, the A2aR-D2 receptor heterodimer theory helps to explain the lessened addictiveness of Captagon versus Amphetamine.

Furthermore, counterfeit Captagon may contain other types of Amphetamines (3,4-methylenedioxymethAmphetamine-MDMA, 3,4-MethylenedioxymethAmphetamine-ecstasy).

On one hand, Amphetamine, the main Captagon metabolite, is an addictive CNS stimulator. On the other hand, Theophylline, another Captagon metabolite that blocks A2aR in the brain, may attenuate the behavioral sensitization caused by Amphetamine. These metabolites make Captagon more potent but less addictive than Amphetamine. The further experiment is on-going, and the data will be published elsewhere.

## Conclusion

In our present work, we systematically analyzed the addiction mechanism of Captagon and its metabolites, Amphetamine and Theophylline, using our established drug abused chemogenomics knowledgebase systems pharmacology methods. Our data mining analysis of signaling pathways for both Amphetamine and Theophylline, reveals that Amphetamine is the main cause of Captagon addiction and Theophylline can attenuate this behavioral sensitization. In addition, we explored the detailed interactions of Amphetamine and Theophylline with their reported targets using molecular docking studies and molecular dynamics simulation studies. Furthermore, using our established hallucinogen-related chemogenomics knowledgebase and in-house computational chemogenomics tools, we studied the side effects of Amphetamine and Captagon through off-target prediction. Overall, such potential drug-drug combinations are a promising method for developing novel medications to treat drug abuse and addiction.

## Electronic supplementary material

Insight of Captagon Abuse by Chemogenomics Knowledgebase-guided Systems Pharmacology Target Mapping Analyses
