# Supplementary material for: Insight of Captagon Abuse by Chemogenomics Knowledgebase-guided Systems Pharmacology Target Mapping Analyses
Source: Sci Rep. 2019 Feb 19;9:2268. doi: 10.1038/s41598-018-35449-6 (PMC6381188; doi:10.1038/s41598-018-35449-6)
Supplement: Supplementary file 1 — Insight of Captagon Abuse by Chemogenomics Knowledgebase-guided Systems Pharmacology Target Mapping Analyses [file 41598_2018_35449_MOESM1_ESM.docx]

**Insight of Captagon Abuse by Chemogenomics Knowledgebase-guided System Pharmacology Target Mapping Analyses**

**Nan Wu^1,2,3,4#^, Zhiwei Feng^1,2,3,4#^, Xibing He^1,2,3,4^, William Kwon^1,2,3,4^ Junmei Wang^1,2,3,4^* and Xiang-Qun Xie^1,2,3,4^***

^1^Department of Pharmaceutical Sciences and Computational Chemical Genomics Screening Center, School of Pharmacy; ^2^National Center of Excellence for Computational Drug Abuse Research; ^3^Drug Discovery Institute; ^4^Departments of Computational Biology and Structural Biology, School of Medicine, University of Pittsburgh, Pittsburgh, Pennsylvania 15260, United States.


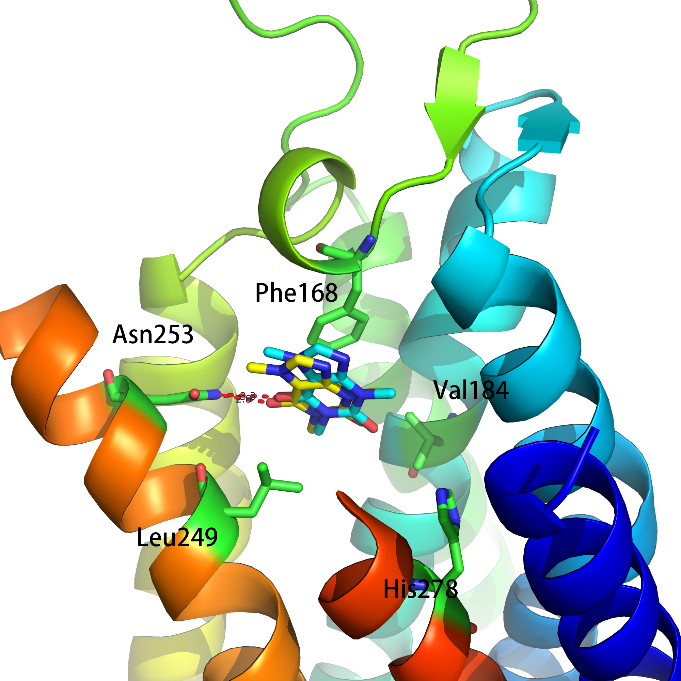

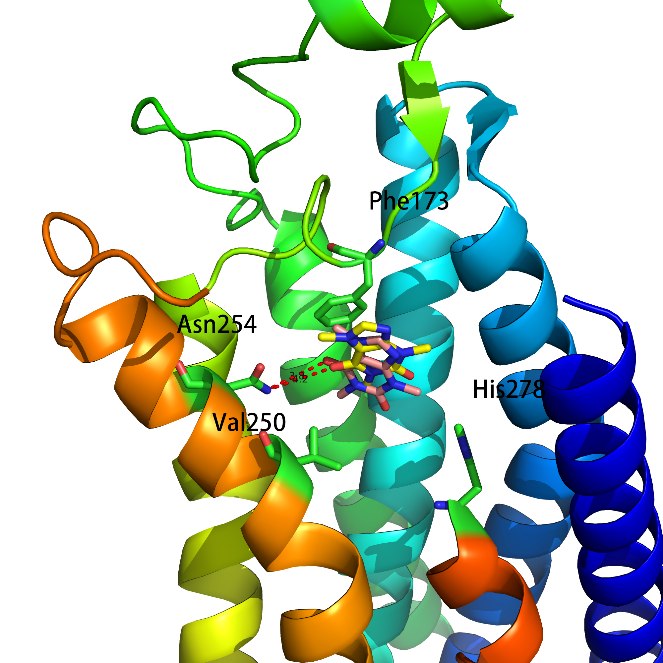


(a) (b)

**Figure S1. Detailed docking interactions of A2a receptor and A2b receptor**. (a). Re-docking of Caffeine back into A2a receptor for the validation of our docking protocol. (b). Docking of Theophylline (red)/Caffeine (yellow) within A2b receptor.
